# Role of inflammation in the initiation and maintenance of atrial fibrillation and the protective effect of atorvastatin in a goat model of aseptic pericarditis

**DOI:** 10.3892/mmr.2014.3116

**Published:** 2014-12-18

**Authors:** YE ZHANG, YU-TANG WANG, ZHAO-LIANG SHAN, HONG-YANG GUO, YUAN GUAN, HONG-TAO YUAN

**Affiliations:** 1 Department of Cardiology, Chinese PLA General Hospital, Haidian, Beijing 100853, P.R. China; 2 The First Department of Cardiology, Chinese PLA General Hospital, Haidian, Beijing 100853, P.R. China

**Keywords:** atrial fibrillation, sterile pericarditis model, inflammation, electrophysiological mechanism, atorvastatin

## Abstract

The present study was designed to determine the association between atrial fibrillation (AF) and inflammation in a goat sterile pericarditis model and to assess the effect of atorvastatin, a cholesterol-reducing drug, on AF. A total of 15 adult male goats were randomly divided into control, untreated pericarditis and atorvastatin-treated pericarditis groups. Pericarditis was induced via thoracotomy and atorvastatin was administered orally (60 mg/day) to the goats in the latter group for the duration of the study, commencing 1 week prior to surgery. The levels of high-sensitivity C-reactive protein (hs-CRP), interleukin(IL)-6 and tumor necrosis factor-α (TNF-α) were significantly elevated following surgery in the untreated pericarditis and atorvastatin groups compared with the control group (P<0.05). However, lower levels of hs-CRP, IL-6 and TNF-α were observed in the atorvastatin group compared with the untreated pericarditis group (P<0.05). Additionally, the animals in the atorvastatin-treated pericarditis group had a longer effective refractory period (ERP) and a higher rate adaptation of the ERP compared with those in the untreated pericarditis group (P<0.05). There was a significant negative correlation between the levels of ERP and hs-CRP in the untreated pericarditis group. The inducibility of AF in the left atrium and the duration of AF in the untreated pericarditis and atorvastatin-treated groups increased significantly following surgery (P<0.05). The pericarditis group, however, had a longer duration of AF compared with the atorvastatin group (P<0.05). Thus, inflammation may promote AF by shortening atrial ERP and by reducing the rate adaptation of ERP. These results suggested that atorvastatin can attenuate AF by inhibiting inflammation and may assist in preventing the occurrence and recurrence of AF following cardiac surgery.

## Introduction

Atrial fibrillation (AF) is the most commonly observed form of clinical arrhythmia and its incidence increases with age (1). AF may predispose patients to thrombosis and myocardial ischemia induced by heart failure, which in turn can trigger malignant arrhythmia events, including ventricular tachycardia and ventricular fibrillation (1). Therefore, investigating the pathogenetic mechanisms underlying AF and developing effective prevention and treatment is of paramount clinical significance. Furthermore, there is currently no widely accepted treatment for AF, highlighting the requirement for more robust and universally applicable treatment strategies for AF.

In recent years, a number of studies have reported that inflammation has a central and positive role in the etiology of AF (2–7), which develops in 25–40% of patients undergoing cardiac surgery (2). The levels of pro-inflammatory interleukin (IL)-6 have been observed to peak 6 h after surgery, whereas C-reactive protein (CRP) and CRP-complement complexes peaked 2 and 3 days after surgery, respectively (2). The AF events were predominantly observed between 2 and 3 days after surgery, a correlation that suggests inflammation may be involved in AF in these patients. Consistent with this hypothesis, a previous study observed that the levels of IL-6 were significantly elevated in patients who developed AF following cardiac surgery (3).

Previous reports have demonstrated that statins or anti-inflammatory hormones inhibit inflammation and limit the development of AF (8–12). Simvastatin inhibits atrial electrical remodeling, which may be associated with its ability to limit inflammatory responses (8). In a canine sterile pericarditis model of AF, Kumagai *et al* (9) demonstrated that atorvastatin reduced the elevation of CRP caused by aseptic pericarditis and reduced the inducibility of AF (9). Notably, anti-inflammatory therapy can effectively prevent the occurrence of postoperative AF in patients with cardiac surgery (10) and these hormones can reduce CRP levels and prevent recurrence of AF (11). Following electrical cardioversion in cases of persistent AF, statins significantly reduce the rate of recurrence of AF (12). Despite this correlative data, however, it remains to be elucidated whether inflammation is directly involved in the pathogenesis of AF. Therefore, improving understanding is important for designing and implementing more effective therapeutic strategies for patients with AF.

The prevalent aseptic pericarditis and goat rapid atrial pacing models (13,14) for investigating AF have potential limitations. In the aseptic pericarditis model, inflammation always precludes AF, thus biasing against the observation of an association between AF and inflammation (13). In the goat rapid atrial pacing model, AF is induced through pacing-induced changes in cardiac electrophysiological characteristics (14); however, this does not enable assessment of the role of inflammation in the process. Thus, in the present study, a goat aseptic pericarditis model that causes rapid atrial excitement was established. A combination of aseptic pericarditis with atrial excitement provides a physiological context to investigate the contribution of the changes in inflammatory cytokines and atrial electrophysiological properties and provides additional insight into the role of statins in the etiology of AF.

## Materials and methods

### Reagents and equipment

Sodium pentobarbital powder was purchased from Sino Pharm Chemical Reagent Beijing Co., Ltd. (Beijing, China). Goat serum hs-CRP, goat serum IL-6 and goat serum tumor necrosis factor (TNF)-α enzyme-linked immunosorbent assay (ELISA) kits were purchased from SunBio Biomedical Technology Co., Ltd. (Beijing, China). Atorvastatin calcium tablets were purchased from Pfizer (Dalian, China). An electrophysiological recording system (cat. no. GY-6328) was purchased from HuaNan Medical Science and Technology Co., Ltd. (Henan, China). A TECS II type program stimulator and Siemens-SV 900C ventilator were purchased from Medico (Padua, Italy) and Siemens (Erlangen, Germany), respectively. Ethicon medical sutures were purchased from Johnson & Johnson (New Brunswick, NJ, USA).

### Animals

Healthy adult male goats (n=15) weighing 20–25 kg were maintained in the PLA General Hospital Experimental Animal Center (Haidian, China), with access to food and water *ad libitum*. The animals were housed in separated large pens with straw bedding and controlled temperature at 22°C. All animal procedures were performed in compliance with the Institutional Animal Care and Use Committee of the PLA General Hospital. The present study was approved by the ethics committee of PLA General Hospital.

### Induction of aseptic pericarditis

The epicardial electrodes were prepared, as described in a previous study (15). Briefly, 1 mm diameter silver wire was used for preparation of the electrode tip, which was 1.5 mm in diameter. The left and right atrial electrode sheets contained five and two pairs of electrodes, respectively. The internal spacing and the distance between the electrodes was 5 mm.

Following a 24 h fast, the goats were administered with intravenous (i.v.) injection of 3% sodium pentobarbital (30 mg/kg) for anesthesia, with additional sodium pentobarbital (10 mg/kg) injected hourly. Ventilator-assisted breathing was provided following right lateral endotracheal intubation. An incision was made at the fourth intercostal space. The pericardium was opened and the heart was exposed. Electrodes were sutured to the free wall epicardium of both atria. Sterile talc powder (5–8 g; AppliChem, Inc., Beijing, China) was used to cover the surface of the atria, which was then covered with a layer of gauze. The control group received electrode implantation only, without talc. The chest was closed layer by layer and a drummed lung ventilator was used to prevent pneumothorax. The electrode wiring was run to the neck skin through a subcutaneous tunnel. Infection was prevented by a twice-daily i.v. infusion of 4,800,000 units of penicillin sodium, commencing 1 day prior to surgery and continuing for 3 days thereafter. Following surgery, the goats were housed in their original cages. Blood samples were harvested at baseline, 12, 24, 48 and 72 h, and 7, 14 and 21 days. Electrophysiological recordings were performed at baseline, 24, 48 and 72 h, and 7, 14 and 21 days.

### Experimental animal grouping and parameter measurements

A total of 15 goats were randomly divided into the following three groups, each containing five animals: No induction of pericarditis (control group); pericarditis induction without statin intervention (subsequently referred to as the pericarditis group) and pericarditis induction with statin intervention (statin group). The animals in the statin group were orally administered with 60 mg atorvastatin calcium daily, commencing 1 week prior to surgery until the experimental endpoint. All the animals underwent thoracotomy, in which atrial epicardial electrodes were implanted. Pericarditis was induced in the pericarditis and statin groups only, as described above. The left and right atrial effective refractory periods (ERP), rate of ERP adaptation, left and right atrial conduction velocity (CV) and AF inducibility and duration were measured in all the animals. The inflammatory markers were assayed from jugular venous blood samples, which were obtained prior to surgery and at the indicated time points following the procedure for up to 21 days. The blood samples were centrifuged at 2,500 × g for 20 min at 4°C. The serum was collected and stored at −80°C. The serum levels of hs-CRP, IL-6 and TNF-α were measured via solid-phase ELISA.

### Tissue specimen preparation and staining

The animals were sacrificed by bleeding after induction of general anesthesia. The left and right atrial free wall tissues were harvested post-sacrifice and fixed in 10% neutral formalin solution for 24 h. Tissue processing and hematoxylin and eosin (H&E) staining was then performed according to standard techniques.

### Statistical analysis

All statistical analyses were performed using SPSS 11.0 software (SPSS, Inc., Chicago, IL, USA). The data are presented as the mean ± standard deviation and were compared using one-way analysis of variance (ANOVA). The inducibility of AF was analyzed using a χ^2^ test. P<0.05 was considered to indicate a statistically significant difference.

## Results

### Atorvastatin reduces inflammatory markers following pericarditis

**Figure 1:**
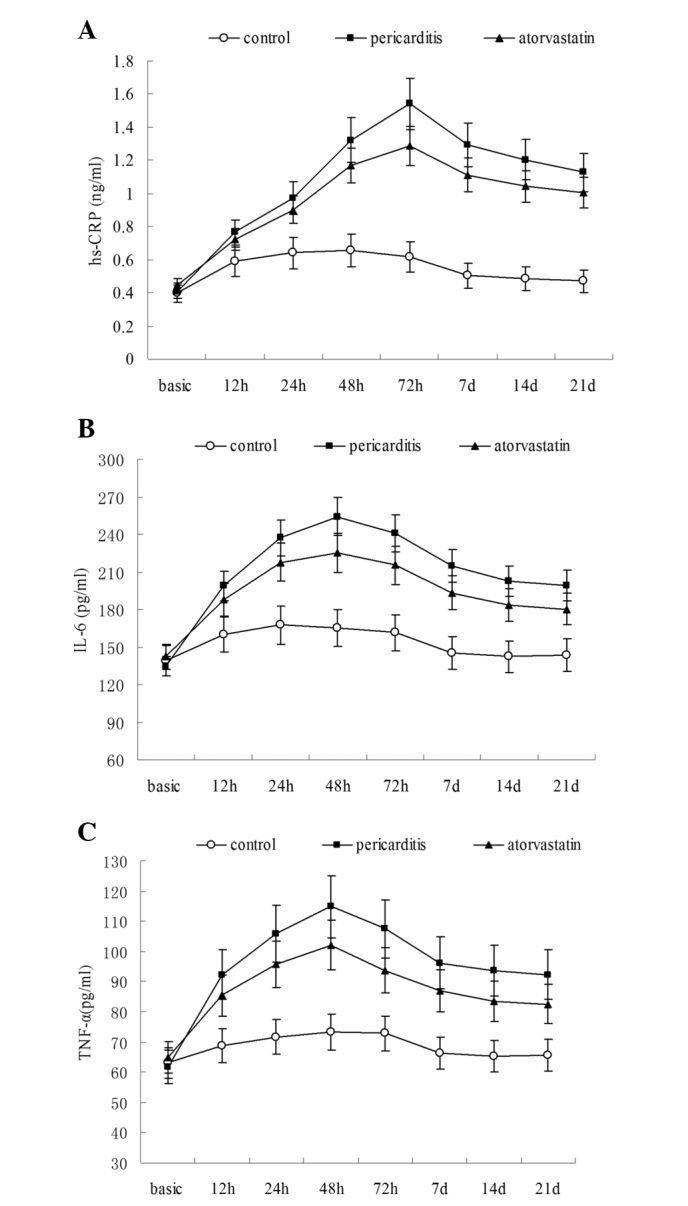
Comparison of the changes in levels of (A) hs-CRP, (B) IL-6 and (C) TNF-α in different experimental groups (n=5/group). Values are presented as the mean ± standard deviation. hs-CRP, high-sensitivity C-reactive protein; IL-6, interleukin-6; TNF-α, tumor necrosis factor-α.

In the preoperative baseline state, no statistically significant difference was observed in the serum levels of hs-CRP, IL-6 or TNF-α between the control, pericarditis and statin groups (Tables I–III). The serum levels of hs-CRP in the statin and pericarditis groups were significantly increased above the baseline levels 12 h after surgery (P<0.05) and peaked 72 h after surgery (1.542±0.114 and 1.287±0.091 ng/ml, respectively). Furthermore, these numbers were significantly higher when compared with the control group (P<0.05; Table I). The levels of hs-CRP began to decline 7 days after surgery, however, they remained significantly elevated compared with the preoperative state (P<0.05; Table I). Notably, the serum level of hs-CRP in the atorvastatin group 48 h after surgery was significantly lower compared with that of the pericarditis group (P<0.05; Table I; Fig. 1A).

In the atorvastatin and pericarditis groups, the serum levels of IL-6 and TNF-α were significantly increased 12 h after surgery (P<0.05), peaked 48 h after surgery and were significantly higher compared with the levels in the control group (P<0.05; Tables II and III; Fig. 1B and C). As with the levels of hs-CRP, the serum levels of IL-6 and TNF-α in the control group began decrease significantly 7 days after surgery and continued to decline until the end of the experiment. At the experiment endpoint, IL-6 and TNF-α remained increased compared with the preoperative baseline level, however, the difference was not statistically significant. In the atorvastatin and pericarditis groups, serum levels of IL-6 and TNF-α gradually decreased, but remained higher than at the preoperative stage, even following the 21 day endpoint (P<0.05). The levels of both cytokines were significantly lower in the atorvastatin group compared with the pericarditis group 24 h after surgery (P<0.05; Tables II and III; Fig. 1B and C).

### Atorvastatin extends left atrial ERP following pericarditis

As expected, the preoperative basic cycle lengths (BCL) at 500, 400, 300 and 200 ms were not significantly different between the three groups (Tables IV–VII). In all groups, the left atrial ERP was significantly lower immediately following surgery compared with the preoperative baseline (P<0.05) and was gradually decreased until 72 h after surgery (Tables IV–VII). The animals in each group exhibited extended left atrial ERP 7 days after surgery, whereas no significant differences in left atrial ERP were observed in the control group at 14 and 21 days compared with the baseline ERP. However, in the atorvastatin and pericarditis groups, the left atrial ERP remained significantly lower than the baseline (P<0.05; Tables IV–VII). Compared with the control group, the left atrial ERP in the pericarditis group was significantly shorter (P<0.05), while in the atorvastatin group, it was significantly prolonged relative to the pericarditis group (P<0.05). No differences were observed in right atrial ERP in the preoperative state, however, between 24 and 72 h postoperatively, the right atrial ERP in the pericarditis group was significantly shorter compared with the control group (P<0.05). The right atrial ERP in each group was significantly longer than the left atrial ERP (P<0.05; Tables IV–VII).

### Atorvastatin assists in the recovery of atrial ERP rate adaptation following pericarditis

**Figure 2:**
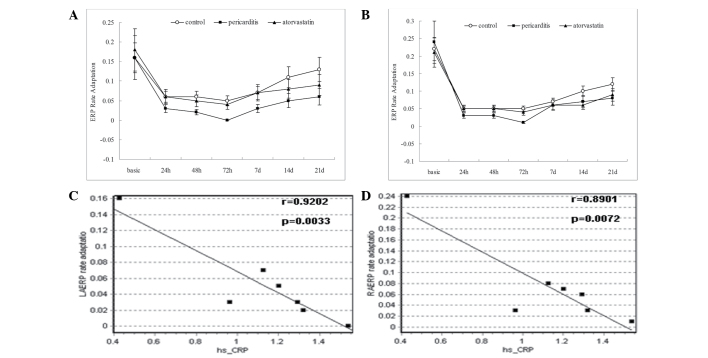
Comparison of changes in the ERP rate adaptation of the (A) LA and (B) RA of each experimental group. Linear regression of the association between the ERP rate adaptation and hs-CRP in the (C) LA and (D) RA of the pericarditis group. Values are presented as the mean ± standard deviation. ERP, effective refractory period; LA, left atrium; RA, right atrium.

The atrial ERP rate adaptation did not differ significantly between the groups in the preoperative state (Table VIII). However, the atrial ERP rate adaptation 24 h after surgery was significantly lower in each group (P<0.05). In the pericarditis group, the left and right atrial ERP rate adaptation 72 h after surgery declined to 0.00±0.02 and 0.01±0.02 ms, respectively, with complete loss of atrial ERP rate adaptation. Following this time point, the atrial ERP rate adaptation of each group gradually recovered. In the control and atorvastatin groups, the left and right atrial ERP rate adaptation had recovered to normal values by the end of the experiment, however, the pericarditis group exhibited poor atrial ERP rate adaptation, which remained significantly lower than baseline levels (P<0.05; Table VIII; Fig. 2A and B).

### Atrial ERP rate adaptation is negatively correlated with serum levels of hs-CRP

Linear regression analysis between the left and right atrial ERP rate adaptations and levels of hs-CRP in the pericarditis group revealed that the left (Fig. 2C) and right (Fig. 2D) atrial ERP rate adaptation and the levels of hs-CRP were negatively correlated (multiple correlation coefficients of 0.9202 and 0.8901).

The left and right atrial conduction velocity (CV) did not vary significantly between the experimental groups. In the preoperative state, no significant difference was observed in atrial CV between the left and right atria in the groups. The atrial CV in each group decelerated postoperatively, however, not significantly compared with the baseline values.

### Atorvastatin reduces the duration, but not the inducibility, of AF following pericarditis

**Figure 3:**
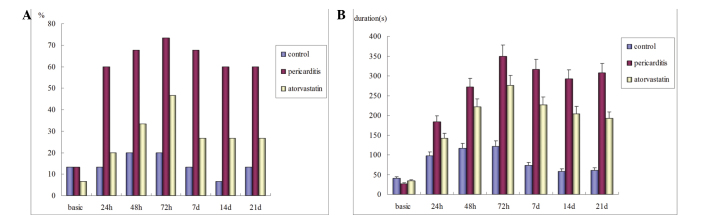
(A) Percentages of left atrium fibrillation inducibility between the different groups. (B) Comparison of goat fibrillation duration between the different groups. Values are presented as the mean ± standard deviation.

Preoperatively, the inducibility of left AF in the control, statin and pericarditis groups were 13.3, 13.3 and 6.7%, respectively (P>0.05). No spontaneous postoperative AF was observed in any of the groups. In the control group, the postoperative left AF inducibility increased marginally compared with the preoperative level, however, the difference was not statistically significant. In the atorvastatin and pericarditis groups, the left AF inducibility was significantly higher compared with the baseline (P<0.05), peaking 72 h after surgery. However, the difference between the two groups was not statistically significant. Additionally, no significant difference was observed in the postoperative right AF inducibility between the three groups (P>0.05; Table IX; Fig. 3A). The durations of postoperative AF in the atorvastatin and pericarditis groups were significantly prolonged compared with the preoperative levels (P<0.05) and were significantly longer compared with the control group (P<0.05). The postoperative AF duration was significantly shorter in the atorvastatin group compared with the pericarditis group (P<0.05; Fig. 3C).

### Comparison of the pathological changes in the different experimental groups

**Figure 4:**
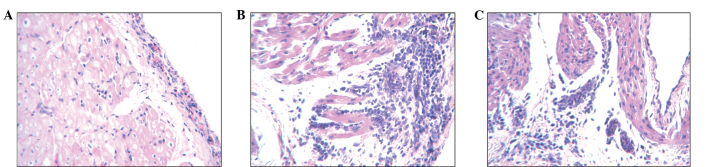
(A) H&E staining of the atrial tissues obtained from the control group epicardium exhibited a low level of inflammatory cell infiltration, but generally normal myocardial cells. (B) H&E staining of the atrial tissues obtained from the pericarditis group had epicardial thickening, infiltration of lymphocytes, myocardial rupture and necrosis. (C) H&E staining of the atrial tissues obtained from statin intervention group had epicardial thickening, but moderate levels of lymphocyte infiltration and myocardial sarcoplasmic cohesion. Magnification, ×200. H&E, hematoxylin and eosin.

H&E staining revealed different levels of visible atrial tissue inflammation in the three groups (Fig. 4). The atrial epicardium in the control group sections exhibited a low level of inflammatory cell infiltration, however the cardiomyocytes were generally normal (Fig. 4A). The pericarditis group tissue exhibited signs of epicardial thickening, infiltration of lymphocytes, myocardial rupture and necrosis (Fig. 4B). The atorvastatin group also exhibited epicardial thickening, however, only moderate levels of lymphocyte infiltration and myocardial sarcoplasmic condensation were observed (Fig. 4C).

## Discussion

Since the initial suggestion of an association between inflammation and AF in 1997 (2), a number of clinical studies have examined the possible links between the two, however, an association between inflammation and AF, and the role of statins in treating AF remain to be elucidated.

At present, there are several animal models of AF, including vagus nerve stimulation (16), sterile pericarditis (13), chronic mitral regurgitation (17), rapid atrial/ventricular pacing (14,18) and hyperthyroidism (19). In the sterile pericarditis model, aseptic inflammation is caused by surgical procedures through AF stimulation. In the present study, this model was selected to induce inflammation and examine the association between inflammation and AF.

Significant increases were observed in the serum levels of hs-CRP, IL-6 and TNF-α 12 h after surgery in the atorvastatin and pericarditis groups, suggestive of an inflammatory response and demonstrating that the sterile pericarditis model in goats induced a distinctive and desirable inflammatory response. In the control group, the serum levels of inflammatory factors also exhibited an initial significant increase, possibly due to the thoracotomy wound. Importantly, however, these molecules decreased to preoperative levels within 14–21 days after surgery.

In the present study, postoperative atrial ERP in the pericarditis group was significantly shorter 2–3 days after surgery, compounded with simultaneous increases of inflammatory cytokines. Following this time point, with the reduction of inflammation and decreased levels of inflammatory factors, atrial ERP did not decrease further and was found to extend, however, it remained significantly shorter compared with the control group. This suggested that the inflammation associated with atrial ERP led to shortened atrial ERP up to 7 days after surgery. In addition, as with the aforementioned inflammatory response, the atrial ERP rate of adaptation gradually declined. Furthermore, at the peak period of the inflammatory response atrial, ERP rate adaptation was lost. Through linear correlation analysis, a negative correlation was identified between the atrial ERP rate adaptability and the serum levels of hs-CRP. In addition, atrial CV did not change significantly following aseptic pericarditis, indicating that inflammation has no impact on CV. This finding is in contrast with the results from a canine sterile pericarditis model reported by Kumagai *et al* (9), although the difference may be due to a lower density of electrodes in the previous study, reducing the ability to accurately measure intra-atrial conduction time.

In the goat aseptic pericarditis model used in the present study, spontaneous AF was observed postoperatively in the goats from each group. AF was further induced by programmed stimulation or burst stimulation, suggesting that, while inflammation caused certain electrophysiological changes in the goat atria, additional predisposing factors were required to consistently trigger AF. Therefore, inflammation had an effect on atrial electrophysiological characteristics, shortened atrial ERP and reduced ERP rate adaptation. Together, these data suggested that the inflammatory processes can form an ‘AF matrix’, in which AF may be more easily induced and maintained in the presence of the appropriate predisposing factors.

Several studies have demonstrated that during the inflammatory response involved in atrial structural remodeling in patients with AF, widespread inflammatory infiltration, myocardial necrosis and interstitial fibrosis are observed in the affected atrial tissue (2–7). This suggests that inflammation can lead to atrial structural remodeling, making it a factor in development and maintenance of AF. A variety of mechanisms underlying the affect of inflammation on atrial structural remodeling have been reported (20). Inflammation can produce TNF-α-induced expression of connective tissue growth factor, thereby inducing myocardial interstitial fibrosis (6). This leads to atrial structural remodeling with subsequent deposition of excess collagens and fibronectin, ultimately resulting in separation among myocardial cells and impaired cell conduction.

Hydroxymethyl glutaryl coenzyme A reductase inhibitors (statins) inhibit cholesterol biosynthesis and are associated with cardiovascular protective effects, which has led to their wide use in clinical settings. In recent years, a growing number of clinical studies have suggested that statins exert an anti-AF effect (21–26). It has also been demonstrated that statins can reduce the expression of inflammatory mediators, including IL-6, TNF-α, CRP and cyclooxygenase (27,28). In the present study, the levels of hs-CRP, IL-6 and TNF-α in the atorvastatin group were significantly lower compared with those in the postoperative group, indicating that atorvastatin significantly reduced the serum levels of inflammatory cytokines in the goat aseptic pericarditis model. H&E staining revealed that the pericarditis group exhibited extensive atrial inflammatory infiltration, whereas only a moderate level was observed in the atorvastatin group. These finding suggested that atorvastatin reduced the extent of atrial muscle inflammation. Compared with the pericarditis group, the atorvastatin group exhibited a significantly prolonged atrial ERP, increased AF inducibility and shorter AF duration, suggesting that atorvastatin inhibited postoperative atrial electrophysiological changes in the model used. Together, these findings indicated that atorvastatin inhibited the atrial electrophysiological changes and contingent structural changes induced by inflammatory response, thereby reducing the inducibility and duration of AF.

A potential limitation of the present study was the lack of multi-site detection on the whole atrial epicardium and on the pulmonary vein, meaning that the full picture of atrial activation was not determined. This reduced the ability to further analyze the role of the atria and the pulmonary vein in AF induced by sterile pericarditis.

The present study demonstrated that inflammation is important in the initiation and maintenance of AF. Inflammation may promote AF by shortening atrial ERP and ERP rate adaptation. Additionally, elevation in the levels of hs-CRP, IL-6, TNF-α induced by atrial tachyrhythmia suggested that AF may promote the inflammatory processes. It is possible that atorvastatin inhibited the atrial electrophysiological changes and contingent structural changes induced by the inflammatory response, thereby reducing the inducibility and duration of AF. Together, these results suggested a potential role for atorvastatin in reducing the incidence of AF following cardiac surgery.

## Figures and Tables

**Table I:** Serum levels of high-sensitivity C-reactive protein in different experimental groups.

Time period	Control group (ng/ml)	Pericarditis group (ng/ml)	Statin intervention group (ng/ml)
Preoperative	0.401±0.036	0.407±0.055	0.445±0.051
Postoperative			
12 h	0.588±0.022^a^	0.766±0.063^a^,^b^	0.724±0.053^a^,^b^
24 h	0.612±0.072^a^	0.974±0.075^a^,^b^	0.903±0.067^a^,^b^
48 h	0.647±0.051^a^	1.323±0.107^a^,^b^	1.172±0.084^a^,^b^,^c^
72 h	0.569±0.047^a^	1.542±0.114^a^,^b^	1.287±0.091^a^,^b^,^c^
7 days	0.504±0.049^a^	1.294±0.105^a^,^b^	1.113±0.075^a^,^b^,^c^
14 days	0.486±0.068	1.204±0.086^a^,^b^	1.042±0.074^a^,^b^,^c^
21 days	0.471±0.072	1.127±0.096^a^,^b^	1.007±0.063^a^,^b^,^c^

Data are expressed as the mean ± standard deviation. n=5 in each group.

a P<0.05, compared with baseline;

b P<0.05, compared with the control group;

c P<0.05, compared with the pericarditis group.

**Table II:** Serum levels of interleukin-6 in different experimental groups.

Time period	Control group (pg/ml)	Pericarditis group (pg/ml)	Statin intervention group (pg/ml)
Preoperative	139.1±14.1	134.7±16.6	142.4±15.2
Postoperative			
12 h	160.2±13.9^a^	198.9±15.7^a^,^b^	187.4±13.3^a^,^b^
24 h	167.6±13.3^a^	237.1±12.3^a^,^b^	217.8±15.3^a^,^b^,^c^
48 h	165.2±16.4^a^	254.2±11.5^a^,^b^	225.5±17.2^a^,^b^,^c^
72 h	161.4±14.7^a^	240.9±12.8^a^,^b^	215.4±16.1^a^,^b^,^c^
7 days	144.8±15.7	215.0±15.2^a^,^b^	193.1±15.8^a^,^b^,^c^
14 days	142.3±17.1	202.7±14.9^a^,^b^	182.3±15.8^a^,^b^,^c^
21 days	143.4±15.5	199.2±13.5^a^,^b^	180.2±13.2^a^,^b^,^c^

Data are expressed as the mean ± standard deviation. n=5 in each group.

a P<0.05, compared with baseline;

b P<0.05, compared with the control group;

c P<0.05, compared with the pericarditis group.

**Table III:** Serum tumor necrosis factor-α levels in different experimental groups.

Time period	Control group (pg/ml)	Pericarditis group (pg/ml)	Statin intervention group (pg/ml)
Preoperative	63.2±6.4	61.9±5.6	65.1±7.4
Postoperative			
12 h	68.9±3.5^a^	92.4±5.7^a^,^b^	85.5±6.1^a^,^b^
24 h	71.7±5.1^a^	105.7±7.4^a^,^b^	94.2±6.4^a^,^b^,^c^
48 h	73.4±7.6^a^	114.8±8.3^a^,^b^	102.1±6.7^a^,^b^,^c^
72 h	72.9±5.5^a^	107.5±10.2^a^,^b^	93.7±7.6^a^,^b^,^c^
7 days	66.3±7.2	96.2±8.9^a^,^b^	86.1±5.8^a^,^b^,^c^
14 days	65.4±6.4	93.6±8.1^a^,^b^	82.4±6.9^a^,^b^,^c^
21 days	65.7±7.3	92.4±7.2^a^,^b^	81.6±7.4^a^,^b^,^c^

Data are expressed as the mean ± standard deviation. n=5 in each group.

a P<0.05, compared with baseline;

b P<0.05, compared with the control group;

c P<0.05, compared with the pericarditis group.

**Table IV:** Comparison of atrial effective refractory period in different experimental groups at a basic cycle length of 500 ms.

	Control group (ms)		Pericarditis group (ms)		Statin intervention group (ms)	
			
	LA	RA	LA	RA	LA	RA
Preoperative	182.7±19.3	228.8±16.0	188.9±21.4	216.9±18.6	184.7±14.1	211.3±12.7
Postoperative						
24 h	151.1±13.4^a^	169.9±11.7^a^	122.3±13.7^a^,^b^	143.2±12.2^a^,^b^	140.7±8.9^a^,^c^	163.6±9.8^a^,^c^
48 h	149.9±9.6^a^	172.2±11.9^a^	123.6±8.7^a^,^b^	141.4±13.2^a^,^b^	135.4±7.3^a^,^b^	160.3±8.0^a^,^c^
72 h	145.9±9.8^a^	170.7±11.8^a^	121.1±11.1^a^,^b^	142.7±19.0^a^,^b^	133.9±7.4^a^	159.6±9.4^a^
7 days	157.4±14.8^a^	190.5±9.3^a^	134.4±17.5^a^,^b^	158.0±27.5^a^	160.7±7.4^a^,^c^	172.6±7.8^a^
14 days	166.3±19.1	196.3±9.6	144.3±8.3^a^,^b^	179.1±18.1^a^	158.0±5.3^a^,^c^	181.7±11.0^a^
21 days	175.1±20.4	219.7±8.5	147.2±12.1^a^,^b^	186.0±15.5^a^,^b^	170.6±7.6^a^,^c^	190.1±13.8^a^,^b^

Data are expressed as the mean ± standard deviation. n=5 in each group. LA, left atrium; RA, right atrium.

a P<0.05, compared with baseline;

b P<0.05, compared with the control group;

c P<0.05, compared with the pericarditis group.

**Table V:** Comparison of atrial effective refractory period in different experimental groups at a basic cycle length of 400 ms.

	Control group (ms)		Pericarditis group (ms)		Statin intervention group (ms)	
			
	LA	RA	LA	RA	LA	RA
Preoperative	176.1±16.4	218.7±17.4	180.3±21.4	210.7±13.9	176.7±13.4	201.1±13.3
Postoperative						
24 h	147.1±16.3^a^	164.6±8.8^a^	113.5±12.5^a^,^b^	138.7±9.8^a^,^b^	131.4±6.3^a^^c^	156.6±14.2^a^,^c^
48 h	145.0±10.8^a^	168.9±10.4^a^	114.1±9.9^a^,^b^	136.8±12.8^a^,^b^	132.2±10.4^a^,^c^	155.9±12.5^a^,^c^
72 h	141.6±13.0^a^	169.9±14.7^a^	112.5±11.1^a^,^b^	136.9±18.6^a^,^b^	129.0±7.5^a^,^c^	154.9±13.4^a^,^c^
7 days	152.7±14.4^a^	187.1±9.0^a^	128.2±19.3^a^,^b^	156.3±26.5^a^	150.2±7.9^a^,^c^	169.1±10.1^a^
14 days	161.3±21.0	192.3±9.3	130.6±15.3^a^,^b^	176.0±15.7^a^	159.9±9.7^a^,^c^	179.3±9.0^a^
21 days	170.6±18.2	212.6±11.1	136.7±15.5^a^,^b^	181.1±13.9^a^,^b^	163.2±9.7^a^,^c^	183.4±11.5^a^,^b^

Data are expressed as the mean ± standard deviation. n=5 in each group. LA, left atrium; RA, right atrium.

a P<0.05, compared with baseline;

b P<0.05, compared with the control group;

c P<0.05, compared with the pericarditis group.

**Table VI:** Comparison of atrial effective refractory period in different experimental groups (mean ± SD, ms) at a basic cycle length of 300 ms.

	Control group (ms)		Pericarditis group (ms)		Statin intervention group (ms)	
			
	LA	RA	LA	RA	LA	RA
Preoperative	164.6±12.2	192.3±12.1	168.3±14.0	192.1±13.6	168.7±9.8	186.6±8.0
Postoperative						
24 h	141.7±17.8^a^	159.7±7.7^a^	105.9±12.1^a^,^b^	132.5±10.2^a^,^b^	124.1±5.1^a^,^b^,^c^	146.7±12.0^a^,^c^
48 h	139.9±13.1^a^	161.2±10.6^a^	106.9±9.3^a^,^b^	134.5±13.7^a^,^b^	121.6±8.3^a^,^b^,^c^	145.3±12.6^a^
72 h	136.5±13.9^a^	165.0±13.5^a^	106.1±10.2^a^,^b^	132.5±16.1^a^,^b^	123.6±7.2^a^,^c^	143.9±11.3^a^
7 days	147±14.6^a^	172.9±9.2^a^	115.1±14.7^a^,^b^	151.8±20.8^a^	134.6±6.1^a^,^c^	162.0±7.3^a^
14 days	152.9±16.0	180.2±7.3	122.5±10.3^a^,^b^	169.7±14.1^a^	144.4±9.8^a^,^c^	169.7±8.5^a^
21 days	156.6±10.8	186.9±11.7	124.7±11.7^a^,^b^	174.7±11.4^a^	149.9±10.2^a^,^c^	174.0±8.2^a^

Data are expressed as the mean ± standard deviation. n=5 in each group. LA, left atrium; RA, right atrium.

a P<0.05, compared with baseline;

b P<0.05, compared with the control group;

c P<0.05, compared with the pericarditis group.

**Table VII:** Comparison of atrial effective refractory period in different experimental groups at a basic cycle length of 200 ms.

	Control group (ms)		Pericarditis group (ms)		Statin intervention group (ms)	
			
	LA	RA	LA	RA	LA	RA
Preoperative	148.8±12.1	170.7±8.5	151.9±11.1	168.4±8.9	150.5±9.1	165.5±7.5
Postoperative						
24 h	135.3±11.9^a^	154.8±10.2^a^	103.3±9.2^a^,^b^	129.1±9.1^a^,^b^	118.4±6.6^a^,^b^,^c^	141.8±10.1^a^
48 h	134.0±12.7^a^	155.9±8.2^a^	104.9±10.2^a^,^b^	131.3±11.9^a^,^b^	116.8±7.1^a^,^b^	140.3±9.7^a^,^c^
72 h	131.4±13.8^a^	160.3±11.9	105.8±10.6^a^,^b^	131.0±17.0^a^,^b^	119.1±8.4^a^	140.3±8.1^a^,^b^
7 days	140.5±12.5	166.6±9.8	111.7±11.4^a^,^b^	146.1±18.9^a^	126.9±4.9^a^,^c^	155.5±7.4^a^
14 days	142.1±10.8	168.3±7.5	117.1±11.7^a^,^b^	162.7±13.0	132.6±6.5^a^,^c^	163.5±6.7
21 days	144.1±11.3	170.5±6.5	118.4±10.9^a^,^b^	167.1±8.9	141.7±8.0^c^	165.0±5.6

Data are expressed as the mean ± standard deviation. n=5 in each group. LA, left atrium; RA, right atrium.

a P<0.05, compared with baseline;

b P<0.05, compared with the control group;

c P<0.05, compared with the pericarditis group.

**Table VIII:** Changes of atrial effective refractory period rate adaptation in the different experimental groups.

	Control group (ms)		Pericarditis group (ms)		Statin intervention group (ms)	
			
	LA	RA	LA	RA	LA	RA
Preoperative	0.16±0.02	0.22±0.11	0.16±0.03	0.24±0.07	0.18±0.06	0.21±0.03
Postoperative						
24 h	0.06±0.02^a^	0.05±0.04^a^	0.03±0.01^a^	0.03±0.03^a^	0.06±0.04^a^	0.05±0.03^a^
48 h	0.06±0.04^a^	0.05±0.04^a^	0.02±0.02^a^	0.03±0.02^a^	0.05±0.02^a^	0.05±0.05^a^
72 h	0.05±0.01^a^	0.05±0.04^a^	0.00±0.02^a^,^b^	0.01±0.02^a^	0.04±0.04^a^,^c^	0.04±0.03^a^
7 days	0.07±0.03^a^	0.06±0.02^a^	0.03±0.04^a^	0.06±0.03^a^	0.07±0.03^a^	0.06±0.04^a^
14 days	0.11±0.06	0.12±0.04^a^	0.05±0.02^a^	0.07±0.02^a^	0.08±0.05^a^	0.06±0.03^a^
21 days	0.13±0.05	0.16±0.12	0.06±0.02^a^	0.07±0.03^a^	0.09±0.06^a^	0.09±0.07^a^

Data are expressed as the mean ± standard deviation. n=5 in each group. LA, left atrium; RA, right atrium.

a P<0.05, compared with baseline;

b P<0.05, compared with the control group;

c P<0.05, compared with the pericarditis group.

**Table IX:** Comparison between the RA and LA percentages of fibrillation inducibility in the different experimental groups.

	Control group (%)		Pericarditis group (%)		Statin intervention group (%)	
			
	LA	RA	LA	RA	LA	RA
Preoperative	13.3	6.7	13.3	0.0	6.7	0.0
Postoperative						
24 h	13.3	13.3	60.0^a^,^b^	13.3	20.0^a^,^c^	13.3
48 h	20.0	13.3	66.7^a^,^b^	20.0	33.3^a^	13.3
72 h	20.0	6.7	73.3^a^,^b^	20.0	46.7^a^,^b^	13.3
7 days	13.3	6.7	66.7^a^,^b^	13.3	26.7^a^,^c^	6.7
14 days	6.7	6.7	60.0^a^,^b^	6.7	26.7^a^	6.7
21 days	13.3	0.0	60.0^a^,^b^	6.7	26.7^a^	6.7

Data are expressed as the mean ± standard deviation. n=5 in each group. LA, left atrium; RA, right atrium.

a P<0.05, compared with baseline;

b P<0.05, compared with the control group;

c P<0.05, compared with the pericarditis group.

## References

[1] Benjamin EJ, Wolf PA, D’Aqostion RB, Silbershatz H, Kannel WB, Levy D (1998). Impact of atrial fibrillation on the risk of death: The Framingham Heart Study. Circulation.

[2] Bruins P, te Velthuis H, Yazdanbakhsh AP (1997). Activation of the complement system during and after cardiopulmonary bypass surgery: postsurgery activation involves C-reactive protein and is associated with postoperative arrhythmia. Circulation.

[3] Gaudino M, Andreotti F, Zamparelli R (2003). The-174 G/C interleukin-6 polymorphism influences postoperative interleukin-6 levels and postoperative atrial fibrillation. Is atrial fibrillation an inflammatory complication?. Circulation.

[4] Frustaci A, Chimenti C, Bellocci F, Morgante E, Russo MA, Maseri A (1997). Histological substrate of atrial biopsies in patients with lone atrial fibrillation. Circulation.

[5] Chung MK, Martin DO, Sprecher D (2001). C-Reactive protein elevation in patients with atrial arrhythmias: inflammatory mechanisms and persistence of atrial fibrillation. Circulation.

[6] Aviles RJ, Martin DO, Apperson-Hansen C (2003). Inflammation as a risk factor for atrial fibrillation. Circulation.

[7] Sata N, Hamada N, Horinouchi T, Amitani S, Yamashita T, Moriyama Y, Miyahara K (2004). C-reactive protein and atrial fibrillation. Is inflammation a consequence or a cause of atrial fibrillation?. Jpn Heart J.

[8] Shiroshita-Takeshita A, Schram G, Lavoie J, Nattel S (2004). Effect of simvastatin and antioxidant vitamins on atrial fibrillation promotion by atrial-tachycardia remodeling in dogs. Circulation.

[9] Kumagai K, Nakashima H, Saku K (2004). The HMG-CoA reductase inhibitor atorvastatin prevents atrial fibrillation by inhibiting inflammation in a canine sterile pericarditis model. Cardiovasc Res.

[10] Yared JP, Starr NJ, Torres FK (2000). Effects of single dose, post induction dexamethasone on recovery after cardiac surgery. Ann Thorac Surg.

[11] Dernellis J, Panaretsu M (2004). Relationship between C-reactive protein concentrations during glucocorticoid therapy and recurrent atrial fibrillation. Eur Heart J.

[12] Siu CW, Lau CP, Tse HF (2003). Prevention of atrial fibrillation recurrence by statin therapy in patients with lone atrial fibrillation after successful cardioversion. Am J Cardiol.

[13] Pagé PL, Plumb VJ, Okumura K, Waldo AL (1986). A new animal model of atrial flutter. J Am Coll Cardiol.

[14] Wijffels MC, Kirchhof CJ, Dorland R (1995). Atrial fibrillation begets atrial fibrillation: A study in awake chronically instrumented goats. Circulation.

[15] Shan ZL, Wang YT, Shi XM (2005). Effects of atrial excitable period on the stability of atrial fibrillation in goats. Zhonghua Xin Xue Guan Bing Za Zhi.

[16] Patterson E, Lu Z, Lin J, Scherlag BJ, Po SS, Coscia D, Lazzara R (2008). Antifibrillatory properties of mivacurium in a canine model of atrial fibrillation. J Cardiovasc Pharmacol.

[17] Kim KH, Kim YJ, Ohn JH (2012). Long-term effects of sildenafil in a rat model of chronic mitral regurgitation: benefits of ventricular remodeling and exercise capacity. Circulation.

[18] Watanabe I, Okumura Y, Kogawa R (2012). Linear catheter ablation of the right atrium for rapid atrial pacing-induced sustained atrial fibrillation in dogs. Int Heart J.

[19] Chen YC, Chen SA, Chen YJ, Chang MS, Chan P, Lin Cl (2002). Effects of thyroid hormone on the arrhythmogenic activity of pulmonary vein cardiomyocytes. J Am Coll Cardiol.

[20] Dernellis J, Panaretou M (2006). Effects of C-reactive protein and the third and fourth components of complement (C3 and C4) on incidence of atrial fibrillation. Am J Cardiol.

[21] Dernellis J, Panaretou M (2005). Effect of C-reactive protein reduction on paroxysmal atrial fibrillation. Am Heart J.

[22] Ozaydin M, Varol E, Aslan SM, Kucuktepe Z, Dogan A, Ozturk M, Altinbas A (2006). Effect of atorvastatin on the recurrence rates of atria fibrillation after electrical cardioversion. Am J Cardiol.

[23] Arribas-Leal JM, Pascual-Figal DA, Tornel-Osorio PL (2007). Epidemiology and new predictors of atrial fibrillation after coronary surgery. Rev Esp Cardiol.

[24] Ozaydin M, Dogan A, Varol E (2007). Statin use before by-pass surgery decreases the incidence and shortens the duration of postoperative atrial fibrillation. Cardiology.

[25] Patti G, Chello M, Candura D, Pasceri V, D’Ambrosio A, Covino E, Di Sciascio G (2006). Randomized trial of atorvastatin for reduction of postoperative atrial fibrillation in patients undergoing cardiac surgery: results of the ARMYDA-3 (Atorvastatin for Reduction of Myocardial Dysrhythmia After cardiac surgery) study. Circulation.

[26] Patel AA, White CM, Shah SA, Dale KM, Kluger J, Coleman CI (2007). The relationship between statin use and atrial fibrillation. CurrMed ResOpin.

[27] Schönbeck U, Libby P (2004). Inflammation, immunity and HMG-CoA reductase inhibitors: statins as antiinflammatory agents?. Circulation.

[28] Mora S, Ridker PM (2006). Justification for the Use of Statins in Primary Prevention: an intervention trial evaluating rosuvastatin (Jupiter) can C-reactive protein be used to target statin therapy in primary prevention?. Am J Cardiol.

